# Timing of complementary feeding is associated with gut microbiota diversity and composition and short chain fatty acid concentrations over the first year of life

**DOI:** 10.1186/s12866-020-01723-9

**Published:** 2020-03-11

**Authors:** Moira K. Differding, Sara E. Benjamin-Neelon, Cathrine Hoyo, Truls Østbye, Noel T. Mueller

**Affiliations:** 1 Department of Epidemiology, Johns Hopkins University Bloomberg School of Public Health, Baltimore, MD USA; 2 Department of Health, Behavior and Society, Johns Hopkins Bloomberg School of Public Health, Baltimore, MD USA; 3 Department of Biological Sciences, North Carolina State University, 3510 Thomas Hall, Raleigh, NC USA; 4 Department of Community and Family Medicine, Duke University, Durham, NC USA; 5 Welch Center for Prevention, Epidemiology and Clinical Research, Johns Hopkins University, 2024 E. Monument St, Suite 2-500, Room 2-521, Baltimore, MD 21205 USA

**Keywords:** Microbiome, Pediatrics, Weaning, Complementary feeding, Butyrate, Metabolites

## Abstract

**Background:**

Early introduction of complementary foods has been associated with various immune disorders, oxidative stress, and obesity in childhood. The gut microbiota and the short chain fatty acids (SCFAs) they produce are postulated to be on the causal pathway. The objective of this study was to determine if early complementary feeding (i.e. consumption of solids or non-water/formula liquids at or before 3 months) is prospectively associated with infant gut microbiota composition, diversity and SCFAs at 3 and 12 months of age in the Nurture birth cohort.

**Results:**

Mother-infant dyads in the early complementary feeding group (*n* = 18) had similar baseline characteristics to those in the later feeding group (*n* = 49). We assessed differential abundance of microbial taxa (measured by 16S rRNA gene sequencing of the V4 region) by timing of complementary feeding using beta-binomial regression models (considering a two-sided FDR corrected *p*-value of < 0.05 as significant), and we fittted linear regression models to assess the association between early complementary feeding and SCFA concentrations (quantified using gas chromatography). After multivariable adjustment for breastfeeding, delivery method, birth weight, and gestational age, there were 13 differentially abundant microbial amplicon sequence variants (ASVs) by timing of introduction to complementary foods at 3 months and 20 ASVs at 12 months. Infants introduced to complementary foods early (vs. later) had higher concentrations of the SCFA butyric acid (mean difference = 0.65, 95% CI: 0.27, 1.04, *p* < 0.01) and total SCFAs (mean difference = 38.8, 95% CI: 7.83, 69.7) at 12 months. *Bilophila wadsworthia* and *Lachnospiraceae Roseburia* were associated with early (vs. later) complementary feeding and with higher butyric acid concentrations at 3 and 12 months, respectively.

**Conclusions:**

Our findings are consistent with the hypothesis that early (vs. later) introduction to complementary foods is associated with altered gut microbiota composition and butyric acid concentrations measured in stool until at least 1 year of age. Further research is needed to determine if these changes mediate future development of metabolic and immune conditions.

## Background

Early introduction to complementary or solid foods in infancy has been associated with increased risk of childhood obesity [1–3], oxidative stress [4], and immune-mediated conditions [5–7], but the mechanism underlying these associations is not yet well understood [6, 8, 9]. It has been postulated that alterations in the infant gut microbiota may be on the pathway [4, 5, 10, 11]. Longitudinal studies of infants have found that adult-associated microbes begin to dominate the infant microbiota after weaning [12, 13], suggesting that early weaning could prematurely displace beneficial infant-associated microbes, altering the metabolic functionality of the gut microbiota during a critical period of development.

Short chain fatty acids (SCFAs) are metabolites derived from the breakdown of indigestible dietary polysaccharides by the colonic microbiota. SCFAs are widely purported to mediate many of the conditions associated with an altered gut microbiota, from inflammation to obesity [14, 15]. When absorbed, SCFAs contribute calories to the host [16], and modulate lipogenesis [17, 18], glycemia [19], and blood pressure [20]. The SCFA butyric acid (i.e. butyrate), in particular, is used as energy by colonocytes to maintain the mucosal barrier [15, 21, 22] and can regulate the expression of genes related to glucose metabolism [23]. Higher concentrations of butyrate and other SCFAs in the stool, however, may indicate that these metabolites are being excreted rather than absorbed [24]. A recent study found that higher SCFAs in the stool, likely driven by gut microbiota dysbiosis, is associated with obesity and hypertension, among other cardiometabolic risk factors [25].

The primary aim of this paper was to examine prospective associations of early introduction to complementary foods with the infant gut microbiota composition and diversity, and fecal SCFA concentrations at 3 and 12 months of age in a longitudinal birth cohort study. We also assessed whether the gut microbiota features that were associated with early complementary feeding correlate with fecal SCFA concentrations. We hypothesized that early complementary feeding (≤ 3 months of age) impacts infant gut microbiota composition and diversity in a manner that results in greater excretion of SCFAs in the stool.

## Results

### Participant characteristics

Of the 70 infants that provided stool at either time point, 3 were missing data on complementary foods, leaving 67 mother-infant pairs in our analytic sample (65 infants had microbiota data at 3 months and 49 at 12 months, with 47 having microbiota data at both time points). Forty two (62.7%) of our analytic sample were of Black or African-American race, 41 (63.1%) were from low-income (≤ $20,000) households, and 46 (68.7%) had overweight or obese mothers. Eighteen (27%) of the infants included were introduced to complementary foods early (≤ 3 months-of-age study visit). At the 3-month study visit, 54 infants (80.6%) were not breastfeeding, 8 (11.9%) were exclusively breastfeeding, and 5 (7.4%) were partially breastfeeding. Infants that were introduced to complementary foods early were similar to those introduced later with respect to baseline characteristics, including initiation and duration of breastfeeding, as shown in Table 1.

**Table 1:** Characteristics of infant-mother dyads in the Nurture cohort with gut microbiota data at 3 or 12 months, stratified by the timing of complementary food introduction

	Early introduction to complementary foods* (*n* = 18)	Later introduction to complementary foods (*n* = 49)	*p*-value
Delivery method (%)			0.63
Vaginal	11 (61.11)	33 (67.35)	
C-Section	7 (38.89)	16 (32.65)	
Pre-pregnancy BMI ≥ 25 kg/m^2^ (%)	14 (77.78)	32 (65.31)	0.33
Maternal age (median [IQR])	25.30 [22.48, 29.09]	26.55 [23.95, 31.97]	0.38
Female sex	8 (44.44)	22 (44.90)	0.97
Infant age at 3 month sample, days (median [IQR])^a^	105.00 [93.25, 117.25]	99.00 [93.00, 107.00]	0.22
Infant age at 12 month sample, days (median [IQR])^b^	374.00 [367.00, 392.00]	371.00 [362.75, 394.50]	0.73
Black or African-American infant race	13 (72.22)	29 (59.18)	0.54
Breastfed ever (%)	14 (77.78)	39 (79.59)	0.87
Breastfeeding duration (weeks), median IQR	4.5 (1.00, 7.00)	4.0 (1.00, 13.04)	0.51
Breastfeeding at time of first sample (%)	2 (11.11)	11 (22.45)	0.30
Maternal low educational achievement (%)	6 (33.33)	25 (51.02)	0.20
Income (%)			0.84
≤ $20,000	11 (61.11)	30 (63.83)	
> $20,000	7 (38.89)	17 (36.17)	
No infant antibiotics at/before 3 months (%)	14 (77.78)	41 (83.67)	0.58
No infant antibiotics at/before 12 months (%)	11 (61.11)	25 (51.02)	0.46
No current maternal smoking (%)^c^	12 (80.00)	36 (80.00)	0.99
Birth weight, kg (median [IQR])	2.94 [2.82, 3.54]	3.23 [2.95, 3.54]	0.52
Gestational age, weeks (median [IQR])	39.93 [37.71, 40.29]	39.29 [38.43, 40.14]	0.87

*Early introduction to complementary foods was defined as introduction of any solids or non-water liquids at or before the 3-month study visit, when stool was collected

^a^Among the 65 infants who provided a sample at 3 months

^b^Among the 49 infants who provided a sample at 12 months

^c^3 infants given solids early and 4 infants given solids later were missing maternal smoking data for at least 1 time point

### Gut microbiota diversity

The estimated mean differences in microbial Shannon diversity associated with early vs. later introduction to complementary foods for both 3- and 12-month old infant fecal samples are summarized in Table 2. Infants introduced to complementary foods early had significantly higher Shannon diversity at both 3 months of age (mean difference = 0.40, 95% CI: 0.25, 0.55) and 12 months of age (mean difference = 0.25, 95% CI: 0.08, 0.42). Timing of introduction to solids (early vs. late) was not significantly associated with Weighted UniFrac at 3 or 12 months of age (see Supplemental Figure 4). Results were robust to covariate adjustment for alternative categorizations of breastfeeding, infant age at stool collection, maternal education, and maternal smoking (see Supplemental Table 3).

**Table 2:** Unadjusted and multivariable-adjusted mean difference in infant gut microbiota Shannon diversity, assessed from infant stool provided at 3 and 12 months of age, according the timing of complementary food introduction

	Infants at 3 months			Infants at 12 months	
	Difference in gut microbiota Shannon diversity (95% CI)			Difference in gut microbiota Shannon diversity (95% CI)	
	Crude Model	Adjusted Model		Crude Model	Adjusted Model
Complementary foods > 3 months (*n* = 47)	0.0 (ref)	0.0 (ref)	Complementary foods > 3 months (*n* = 35)	0.0 (ref)	0.0 (ref)
Complementary foods ≤3 months (*n* = 18)	0.29*** (0.20, 0.37)	0.16** (0.05, 0.28)	Complementary foods ≤3 months (*n* = 14)	0.37* (0.16, 0.58)	0.31* (0.16, 0.46)

Multivariable-adjusted linear models included adjustment for breastfeeding (ever vs. never), delivery mode, gestational age (> median gestational age vs. ≤ median gestational age), and birth weight (> median birth weight vs. ≤ median birth weight)

* = *p* < 0.05, ** = *p* < 0.01, and *** = *p* < 0.001

### Gut microbiota composition

**Fig. 1:**
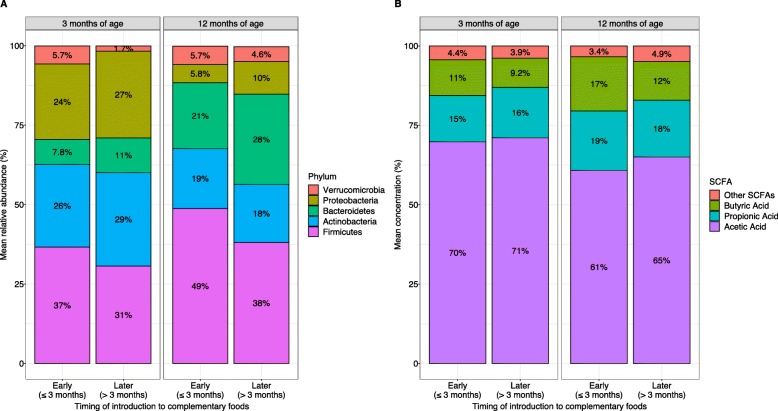
Unadjusted mean percent of **a** major bacterial phyla at 3 and 12 months of age, and **b** major SCFAs at 3 and 12 months of age, stratified by the timing of introduction to complemenatry foods. *Low abundance SCFAs including isobutyric acid, valeric acid, isovaleric acid, heptanoic acid, and hexanoic acid have been grouped into the “other” category for visual clarity

**Fig. 2:**
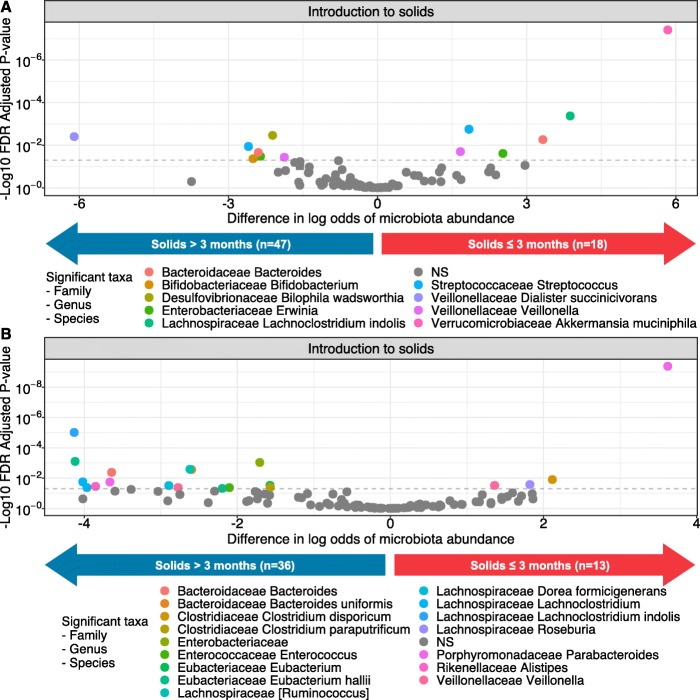
Differences in the log odds of bacterial ASV relative abundance according to the timing of introduction to complementary foods at **a** 3 months of age and **b** 12 months of age. *Associations adjusted for delivery mode, breastfeeding, gestational age, and birth weight. NS = Not significant

The mean relative abundances of major bacterial phyla at 3 and 12 months by timing of introduction to complementary foods are shown in Fig. 1. Early (vs. later) introduction of complementary foods was associated with differential abundance of a total 29 bacterial ASVs in the infant gut at 3 and 12 months of age, after adjustment for potential confounders (Fig. 2 and Supplementary Tables 1 and 2). In the gut microbiota at 3 months of age, early introduction to complementary foods was significantly associated with higher relative abundance of 6 ASVs, including *Akkermansia muciniphilia*, *Lachnoclostridium indolis*, *Bacteroides* (sp. unknown), *Erwinia* (sp. unknown), *Streptococcus* (sp. unknown), and *Veillonella* (sp. unknown), and lower relative abundance of 7 ASVs, including *Veillonella* (sp. unknown), *Bilophila wadsworthia*, *Erwinia* (sp. unknown), *Bacteroides* (sp. unknown), *Bifidobacterium* (sp. unknown), *Streptococcus* (sp. unknown), and *Dialister succinicivorans*.

At 12 months of age, early complementary foods (vs later) was still significantly associated with higher abundance of 4 ASVs, including *Parabacteroides* (sp. unknown), *Clostridium disporicum*, *Roseburia* (sp. unknown), and *Veillonella* (sp. unknown), and lower abundance of 16 ASVs including *Clostridium paraputrificum, Eubacterium* (sp. unknown), *Enterobacteriaceae* (genus and sp. unknown), *Enterococcus* (sp. unknown), 2 ASVs of *Eubacterium hallii*, *Bacteroides uniformis*, *Ruminococcus* (sp. unknown), *Veillonella* (sp. unknown), *Dorea formicigenerans*, *Bacteroides* (sp. unknown), *Parabacteroides* (sp. unknown), *Alistipes* (sp. unknown), 2 *Lachnoclostridium* (sp. unknown), and *Lachnoclostridium indolis.*

### SCFA concentrations

Mean concentrations of fecal SCFAs at 3 and 12 months by timing of introduction to complementary foods are shown in Fig. 1. Univariable and multivariable differences in the fecal concentrations of butyric acid, propionic acid, and acetic acid at 3 months of age according to the timing of complementary foods are shown in Table 3. Early complementary feeding was not associated with fecal butyric acid (mean difference = 0.11 ln-transformed μmol/g, 95% CI: − 0.31, 0.53), propionic acid (mean difference = 0.04 ln-transformed μmol/g, 95% CI: − 0.36, 0.44), or acetic acid (mean difference = − 0.36 μmol/g, 95% CI: − 18.6, 17.9) concentrations in the stool samples collected at 3 months of age.

**Table 3:** Unadjusted and multivariable-adjusted mean difference in short-chain fatty acid concentrations (μmol/g), from infant stool provided at 3 and 12 months of age, according the timing of complementary food introduction

	Butyric Acid^±^		Propionic Acid^±^		Acetic Acid		Total SCFAs	
	Mean difference (95% CI)		Mean difference (95% CI)		Mean difference (95% CI)		Mean difference (95% CI)	
	Crude	Adjusted	Crude	Adjusted	Crude	Adjusted	Crude	Adjusted
Infants at 3 months								
Complementary foods > 3 months (*n* = 47)	0.0 (ref)	0.0 (ref)	0.0 (ref)	0.0 (ref)	0.0 (ref)	0.0 (ref)	0.0 (ref)	0.0 (ref)
Complementary foods ≤3 months (*n* = 18)	0.19 (−0.26, 0.64)	0.11 (−0.31, 0.53)	0.01 (− 0.39, 0.41)	0.04 (− 0.36, 0.44)	0.29 (−17.4, 18.0)	− 0.36 (−18.6, 17.9)	3.88 (−20.2, 27.9)	2.82 (−21.8, 27.4)
Infants at 12 months								
Complementary foods > 3 months (*n* = 36)	0.0 (ref)	0.0 (ref)	0.0 (ref)	0.0 (ref)	0.0 (ref)	0.0 (ref)	0.0 (ref)	0.0 (ref)
Complementary foods ≤3 months (*n* = 13)	0.67*** (0.30, 1.04)	0.65** (0.27, 1.04)	0.35* (0.03, 0.68)	0.31 (−0.03, 0.65)	21.0* (0.65, 41.4)	21.3 (−0.42, 43.0)	40.5** (11.3, 69.8)	38.8* (7.83, 69.7)

^±^Natural log + 1 transformation used for butyric acid and propionic acid at 3 and 12 months

Multivariable-adjusted model includes adjustment for breastfeeding (ever vs. never), delivery mode, gestational age, and birth weight

Total SCFAs includes butyric acid, propionic acid, acetic acid, isobutyric acid, valeric acid, isovaleric acid, heptanoic acid, and hexanoic acid. * = *p* < 0.05, ** = *p* < 0.01, and *** = *p* < 0.001

At 12 months of age, early complementary feeding was still significantly associated with higher fecal butyric acid (mean difference = 0.65 ln-transformed μmol/g, 95% CI: 0.27, 1.04), higher propionic acid (mean difference = 0.31 ln-transformed μmol/g, 95% CI: − 0.03, 0.65), and higher acetic acid (mean difference = 21.3 μmol/g, 95% CI: − 0.42, 43.0) concentrations. In sensitivity analyses, results were similar in models that included alternative categorization of breastfeeding and additional covariates (Supplemental Table 4).

### Correlation between gut microbiota diversity and composition and SCFAs

Shannon diversity at 3 months of age was positively correlated with fecal butyric acid at 12 months of age (*ρ* = 0.31, *p* = 0.04), but not with propionic acid (*ρ* = 0.22, *p* = 0.14) or acetic acid (*ρ* = 0.25, *p* = 0.09). Shannon diversity at 12 months of age was also positively correlated with concentrations of fecal butyric acid (*ρ* = 0.38, *p* = 0.007), as well as propionic acid (*ρ* = 0.40, *p* = 0.004), but not with acetic acid (*ρ* = 0.22, *p* = 0.12) assessed at 12 months of age.

**Fig. 3:**
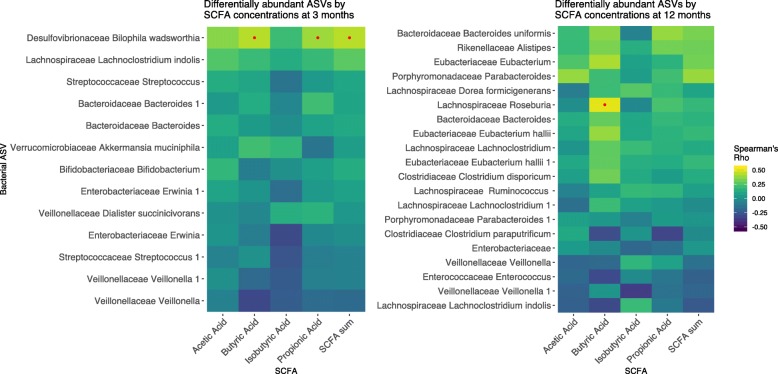
Spearman correlations of the four most abundant fecal SCFAs and total SCFA concentrations with the relative abundances of 3- and 12-month gut microbiota ASVs that were significantly associated with timing of introduction to complementary foods in multivariable-adjusted models. *Yellow indicates a positive correlation, while dark blue indicates a negative correlation. Red dots indicate correlations with FDR-adjusted *p* value < 0.05

Heatmaps of the Spearman correlation coefficients of significant ASVs and SCFA concentrations measured at 3 and 12 months of age are shown in Fig. 3. At 3 months of age, the only significant correlations after FDR correction were *B. wadsworthia* with butyric acid (*ρ* = 0.43, FDR *p* = 0.01), propionic acid (*ρ* = 0.39, FDR *p* = 0.03, and total SCFAs (*ρ* = 0.0.43, FDR *p* = 0.01). At 12 months of age, *L. Roseburia* (sp. unknown) was significantly correlated with butyric acid after FDR correction (*ρ* = 0.51, FDR *p* = 0.02). Other taxa that correlated with butyric acid at 12 months (albeit non-significantly after FDR correction) included *Eubacterium* (sp. unknown) (*ρ* = 0.41, FDR *p* = 0.13), *Eubacterium hallii* (*ρ* = 0.38, FDR *p* = 0.13), *Bacteroides uniformis* (*ρ* = 0.36, FDR *p* = 0.16), *Clostridium disporicum* (*ρ* = 0.31, FDR *p* = 0.21), *Lachnoclostridium indolis* (*ρ* = − 0.31, FDR *p* = 0.22), and *Enterococcus* (sp. unknown) (*ρ* = − 0.30, FDR *p* = 0.22). *Bacteroides uniformis* correlated with propionic acid (*ρ* = 0.38, FDR *p* = 0.13), while *Parabacteroides* (sp. unknown) correlated with acetic acid (*ρ* = 0.38, FDR *p* = 0.13).

## Discussion

In our prospective pre-birth cohort of racially diverse mother-child dyads from North Carolina, we found that early complementary feeding (≤ 3 months of age vs. later) was associated with higher gut microbiome diversity and differential abundance of several critical bacterial taxa at 3 and 12 months of age. Early complementary feeding was also associated with greater concentration of fecal butyrate and total SCFAs at 12 months. Furthermore, gut microbiome diversity measured at 3 months was correlated with SCFA concentrations at 12 months, suggesting that changes in the gut microbiome may precipitate changes in SCFA concentrations.

We are not aware of other prospective studies that have examined the effect of timing of complementary foods on gut microbiota composition, biodiversity, and SCFAs later in life. Our findings appear to be consistent with studies that have assessed how the gut microbiome and SCFA profile changes from pre-weaning to post-weaning. The multinational European INFABIO study (*n* = 605 infants), a case-study by Koenig et al. (*n* = 1 infant), and a small Atlanta-based cohort by Thompson et al. (*n* = 9 infants), found that early introduction of complementary foods was associated with a decrease in *Bifidobacteria* (sp. unknown) and an increase in more adult-associated bacteria, including those from the genus *Bacteroides* and the phylum Verrumicrobia [26–28]. A study by Pannaraj et al. (*n* = 107 infants) on infant diet and the gut microbiome found that introduction to complementary foods before 4 months resulted in a faster maturation in gut microbiome composition than introduction to complementary foods at or after 4 months of age [29]. Our study extends these findings by showing that the impact of early introduction to complementary foods on the gut microbiome may persist beyond the initial microbial transition, until at least 1 year of age.

In addition to changes in gut microbiota composition, we observed an increase in gut microbiota (Shannon) diversity at both 3 and 12 months in infants introduced to complementary foods early. The determinants and consequences of differences in gut microbiome diversity in the first year of life are not well understood. A previous birth cohort study from Canada found that compared to breastfed infants, formula fed infants had greater diversity at 3 months of age, but lower diversity at 1 year [30]. Another study found that higher diversity at 3 months of age is associated with higher risk for overweight later in life [31]. As such, we postulate that higher diversity in early infancy, before the gut microbiome has become adult-like, may reflect a less healthy gut microbiome and portend risk for future development of chronic disease. Larger longitudinal birth cohort studies are needed to test this hypothesis.

Infants introduced to complementary foods early in our study also had significantly higher fecal SCFA concentrations at 12 months. Koenig et al. found that transitioning from breastmilk or formula to complementary foods increased the concentrations of SCFAs [27]. Our study extends this finding, suggesting that the impact of earlier vs. later complementary feeding on SCFA production can be observed out to at least 1 year of age, when all children have begun eating complementary foods. We also extend previous literature by showing that *Bilophila wadsworthia* and *Lachnospiraceae Roseburia* were associated with early complementary feeding and with higher butyric acid concentrations at 3 and 12 months of age, respectively. *L. Roseburia* are known butyrate producers, and, in adults, are postulated to have beneficial effects on the gut mucosa [32] and cardiometabolic health [33]. However, the role of this genus in the health of infants has not been well studied, and may represent accelerated microbiome maturation. *B. wadsworthia* belongs to the Desulfovibrionaceae family, which are known for their sulfate reducing capabilities and have been previously found in healthy infants [34, 35]. Sulfates are found in a variety of infant formulas [36] and breastmilk [37], and so early introduction to complementary foods may reduce dietary sulfate, thus decreasing the abundance of *B. wadsworthia*. We also found that Shannon diversity at 3 months was positively associated with concentration of butyrate at 12 months, suggesting that early impacts microbiome diversity may precipitate later changes to SCFA production.

Our findings may have implications for development of immunometabolic-inflammatory conditions later in life. After adjustment for breastfeeding, early introduction of complementary foods was associated with lower relative abundance of *Bifidobacterium* (sp. unknown) at 3 months of age. Human-associated *Bifidobacterium* spp. are dominant members of the infant microbiome until their decline during weaning [12, 38], and play a critical role by fermenting non-digestible carbohydrates, including those present in breastmilk (oligosaccharides) and some formulas, and producing acetic acid and B vitamins like riboflavin and tetrahydrofolic acid [39–42]. These fermentation by-products can be used by neighboring microbiota to produce butyrate and propionate. Further, a randomized trial (*n* = 90 infants) of infants younger than 7 months with atopic dermatitis in the Netherlands found that supplementing *Bifidobacterium breve* and prebiotics for 12 weeks reduced the incidence of asthma-like symptoms compared to the placebo infants [43]. As such, prematurely reducing the abundance of *Bifidobacterium* by introducing complementary foods too early then could stunt their interaction with the immune system, leading to higher levels of inflammation [40].

Notably, early introduction to complementary foods was associated with markedly higher relative abundance of the mucin-degrading *Akkermansia muciniphila* in the stool collected at 3 months of age. While higher relative abundance of *Akkermansia muciniphila* has been associated with improved metabolic health in adults [44–47], it is possible that this typically beneficial taxa could play an adverse role in infants. Although *Akkermansia* is an efficient degrader of mucin, with the by-products feeding other commensals, it is capable of metabolizing other compounds, leaving dependent taxa to either seek other energy sources or decline in prevalence [48, 49]. Further, the secondary metabolites produced from degrading mucin may simply promote the growth of adult-associated bacteria too early, pushing out more developmentally essential taxa such as *Bifidobacteria* in their stead [12, 26, 50, 51].

Although we found that infants given early complementary foods had higher butyrate concentrations at 12 months of age, we cannot discern whether this is the result of SCFA production vs. excretion, for which there is a measurable trade off [24]. Higher fecal concentrations of butyrate and propionate in humans has been associated with worse metabolic outcomes like obesity and hypertension, while higher serum SCFAs have been associated with better health [25, 52, 53]. Inflammation in the gut could be triggered by the early displacement of infant-associated microbes and metabolites, subsequently reducing the absorption of butyrate and increasing its excretion in the feces [54]. Still, the majority of human studies on SCFAs and immunometabolic conditions have been performed in adults, making it unclear if these relationships hold true for infants [20–22]. As the nutritional requirements for infants change significantly across the first year of life to support rapid growth during this period, there could be differences in the role of the gut microbiota and subsequent production of SCFAs on metabolism [53, 55]. More research is warranted to determine if higher fecal SCFA concentrations in infants are associated with development of health outcomes later in life.

### Strengths and limitations

Our study is strengthened by its longitudinal design, the nuanced infant diet data collected along with detailed covariate data, and the socio-demographic diversity of the Nurture cohort. As a result, we were able to prospectively determine the month of complementary food introduction, extract critical covariate information (e.g. delivery mode and antibiotic use) from electronic medical records, increasing the accuracy of our data and reducing recall bias, and adjust for these covariates in multivariable regression models. Furthermore, by sampling the stool microbiota and SCFAs longitudinally, we were better able to account for technical variation in our data and increase the precision of our associations.

There were several limitations to our study. First, we did not have complete data on specific types of foods consumed at each month for all infants. Among infants who reported the type of solid, the most commonly consumed food for both early and later solids groups from 3 to 6 months of age was infant cereal. At 6 months of age, however, 44/49 infants given solids after 3 months and 11/18 infants given solids at or before 3 months did not report cereal consumption, preventing us from making reliable statistical comparisons between these groups. Also, cereal was sometimes given along with small quantities of other items, such as fruit, vegetables, baby snack foods, or 100% juice. Second, our sample size was too small to stratify on potential effect measure modifiers (e.g. sex, race, breastfeeding duration, and delivery mode), or examine the association of individual foods with microbiota or SCFA outcomes. We also did not anticipate having the statistical power to reliably detect differences in the relative abundance of more rare taxa, so we removed these before testing to reduce the probability of reporting false positives and to avoid inflating the FDR-corrected *p*-values for others. Because this filtering may remove true positives with smaller effect sizes, it is important that larger, future studies investigate these less prevalent taxa. Furthermore, because we compared groups with different sample sizes across time points, we included bootstrap testing when possible to minimize the influence of unbalanced sample size to our findings. Another limitation to our study is that we relied on 16S rRNA gene sequencing data, which did not always allow for species level resolution nor provide us with data on metagenomic functionality. We also did not have blood measurements available to measure circulating SCFAs. In addition, while we cannot rule out confounding by indication, the reasons mothers provided for introducing early complementary foods were quite varied [the top reasons being that their child was hungry (*n* = 3 infants) or that their child was drinking too much formula (*n* = 3 infants)], suggesting that no single factor could explain the associations. Finally, despite extensive covariate adjustment, we cannot exclude the possibility of unmeasured or residual confounding influencing our study results.

## Conclusion

Our study on the timing of the introduction of complementary foods with gut microbiota diversity, composition and fecal SCFA concentrations adds to the literature by showing that early introduction to solids (≤ 3 months) is associated with higher gut microbiota diversity and altered gut microbiota composition at both 3 and 12 months of age and higher fecal butyrate and total SCFAs at 12 months of age, after adjustment for breastfeeding and other potential confounders. The findings described in our study help explain how early introduction to complementary foods may be biologically related to oxidative stress, obesity and immune disorders through changes in the developing infant gut microbiota and associated SCFAs. Larger longitudinal gut microbiota studies that record incident disease, quantify microbiota-associated metabolites, assess microbiota functional content via metagenomic sequencing, and use more sophisticated mediation analyses, are still needed to elucidate whether the microbiome is on the path between the timing of complementary food introduction and later life health conditions.

## Methods

### Study population

We performed our study within the prospective Nurture pre-birth cohort [56]. We recruited women with a singleton pregnancy at 20–36 weeks of gestation from both a county health department and private prenatal clinic in Durham, North Carolina from 2013 to 2015. We required women to be at least 18 years of age, be able to speak and read English, and to confirm their intention to remain local for home visits for at least one year. We further excluded infants born before 28 weeks of gestation, if they had congenital abnormalities, or who required 3 or more weeks of hospitalization after birth. We obtained written informed consent from each mother at recruitment and again after delivery. We performed home visits at 3, 6, 9, and 12 months of age, supplemented by monthly automated interactive voice response (IVR) telephone calls in between visits. We asked a subsample of mothers to collect stool samples for microbiome and SCFA analyses from infants at 3 and 12 months of age. This study was conducted according to guidelines in the Declaration of Helsinki and all procedures involving human subjects were approved by Duke University Medical Center institutional review board (human subjects committee) (Pro0036242). The study is registered at clinicaltrials.gov (NCT01788644).

### Exposure – timing of complementary foods

Mothers reported whether they had introduced complementary foods, the type of foods given, and whether they were still breastfeeding, during monthly IVR calls and home visits at 3, 6, 9, and 12 months of age. We defined “early introduction to complementary foods” as introduction of any solids or non-water liquids or formula at or before the 3-month study visit, when stool was collected. Thus, all associations were prospective in relation to outcomes assessed at both 3- and 12-months. Our categorization of early complementary feeding is in accordance with The American Academy of American Academy of Asthma, Allergy and Immunology [55].

### Covariate measurement

We abstracted data on delivery mode (C-section vs. vaginal delivery, with additional information on type of C-section), birth weight (kg) and length (cm), infant sex, gestational age (weeks), and infant antibiotic use from medical records. We collected maternal age (years), ethnicity and race, pre-pregnancy weight (kg) and height (m), highest education obtained, household income, and smoking status at the time of birth. We used self-reported maternal pre-pregnancy weight and height to calculate pre-pregnancy body mass index (BMI; kg/m^2^), and considered a BMI ≥ 25 as overweight or obese.

### Sample collection and microbial 16S rRNA gene extraction

We collected stool at the 3- and 12-month home visits. Stool was collected from diapers, transferred to a 2 ml cryogenic vial (ThermoFisher), and immediately frozen at − 80 °C for later processing. We thawed frozen specimens and extracted DNA using the QIAgen MagAttract PowerSoil for KingFisher. We deposited 0.5 g of stool in each bead plate well of the PowerSoil kit and extracted DNA per the manufacturer’s instructions. After, we quantified DNA with the Quant-iT dsDNA high sensitivity kit (ThermoFisher).

### 16S rRNA gene sequencing and quality control

We used the Thermo Phusion Hot Start II DNA Polymerase (ThermoFisher) to perform PCR amplification with 1–10 ng of extracted DNA. We started PCR at 98 °C for 2 min, then ran 30 cycles at 98 °C for 20 s, 55 °C for 15 s, and 72 °C for 30 s, finishing at 72 °C for 10 min. We prepared libraries using standard operating procedures described by Kozich et al. [57]. We used 10 μl of PCR product to normalize the SepalPrep Normalization Prep Plate Kit (ThermoFisher Cat. No. A1051001) to 1–2 ng/μl. We pooled 5 μl from each normalized sample into a single library per 96-well plate, then used the DNA Clean and Concentrator kit (Zymo Cat. No. D4013) to concentrate library pools.

For quality control steps, we first performed a dilution series for each pooled library. To determine approximate fragment size and verify library integrity, we analyzed them using the Agilent Bioanalyzer and High Sensitivity DS DNA assay (Agilent Cat. No. 5047–4626). We used the Qiagen QIAquick Gel Extraction Kit (Qiagen Cat. No. 28706) to purify pools with unintended amplicons. We used the KAPA Library Quantification Kit for Illumina (KAPA Cat. No KK4824) to determine pooled library concentrations. We diluted library pools to 4 nM and denatured them with 2.0 N NaOH. We loaded pools at 8 pM with a PhiX spike-in of 20%.

We then performed paired-end sequencing of all samples’ 16S rRNA gene V4 regions with the MiSeq 500 Cycle V2 Reagent Kit (Illumina Cat. No. MS-102-2003) on an Illumina MiSeq. We used the primers and sample-specific barcode demultiplexing described by Kozich et al. [57].

### 16S rRNA gene sequence filtering, denoising, chimera removal, and taxonomic assignment

We used the R package DADA2 (v1.8.0) to perform read quality control and resolve amplicon sequence variants (ASV) as recommended by authors of the package [58]. After viewing sequence quality scores across forward and reverse reads (shown in Figure S1), we trimmed all forward reads before position 10 and after position 240, and all reverse reads before position 10 and after position 150 (5′ and 3′ ends, respectively). We used the DADA2 “filterAndTrim” function to filter out any reads mapping to the phiX genome, reads with ambiguous base assignments, and reads predicted to have more than two expected errors given their quality scores [58].

We randomly selected a subset of 106,431,120 bases from 462,744 reads for the forward error model estimation and 102,199,160 bases from 729,994 reads for the reverse read error model using the “learnErrors” function [58, 59]. We used these error models, which are shown in Figure S2, for denoising on all remaining forward and reverse sample reads.

We dereplicated reads using the “derep” function and denoised these using the “dada” function for pooled reads with default settings and the previously estimated error models. We merged overlapping paired reads using the “mergePairs” function, and used the function “removeBimeraDenovo” to remove estimated chimeras, resulting in a final sequence pool retaining 74% of the raw reads (sequencing step summary shown in Supplementary Figure S3). We assigned taxonomy using the HITdb v.1.00 16S rRNA sequence database of human intestinal taxa and the dada2 function “assignTaxonomy” [60].

### Phylogenetic tree generation

We generated a phylogenetic tree from denoised ASVs following the recommendations in a recently published workflow [59]. Briefly, we used the R package DECIPHER to first align ASVs and construct a neighbor-joining tree [61]. We then used these with the R package phangorn to generate a Generalized time-reversible with Gamma rate variation (GTR) maximum likelihood phylogenetic tree, which we rooted at the midpoint [62]. After, we used the R package phyloseq to merge the phylogenetic tree and ASVs with the sample metadata and taxonomy [63].

### Short chain fatty acid quantification

We quantified SCFA using gas chromatography (Thermo Trace 1310) coupled to a flame ionization detector (Thermo) as previously described [64]. We homogenized stool samples using MP Bio FastPrep after resuspension in MilliQ-grade water at 4.0 m/s for 1 min. We acidified fecal suspensions by adding 5 M HCl until a final pH of 2.0 was reached, then centrifuged at 10,000 RPM (about 9633 g, Sorvall Legend Micro 21R) after incubation. We spiked the supernatants with 2-Ethylbturyic acid until we reached a concentration of 1 mM. We detected SCFAs from the supernatant via direct injection into a Thermo TG-WAXMS A GC Column (30 m, 0.32 mm, 0.25 μm). Injected standard solutions of individual SCFAs were used for calibration. The samples were processed for 16S rRNA sequencing first and then rethawed for gas chromatography.

## Statistical analysis

### Microbial community differential abundance analysis

We used beta-binomial regression models that account for within-sample taxa correlation and variable sequencing depth from the R package corncob (version 3.3.3; *R* Foundation for Statistical Computing, Vienna, Austria) to test for differential abundance of taxa in infants at 3 and 12 months [65]. To avoid testing rare ASVs with our smaller sample size, we removed ASVs that did not have a mean count at or above the 25th percentile in at least 10% of samples.

### Alpha diversity analysis

We used ecological network regression models to estimate Shannon diversity with the R package DivNet [66]. We then tested for differences in Shannon diversity using the hierarchical model Betta, which accounts for incomplete community sampling and allows for multivariable adjustment [67].

### Beta diversity analysis

We used the *R* package phyloseq to estimate weighted UniFrac distances, a measure of pairwise community composition, and performed a Principal Coordinate Analysis to visually assess clustering by variables of interest (Supplemental Figure 4) [63, 68]. We used permutational multivariate analysis of variance (PERMANOVA) as implemented in the *R* package vegan with 9999 permutations to test for differences in weighted UniFrac distances before and after multivariable adjustment (see covariates adjusted for below) [69].

### SCFA regression models and correlations

We fitted univariable and multivariable generalized linear regression models to examine the association of timing of introduction to complementary foods with infant fecal SCFA concentrations (μmol/g) at 3 and 12 months of age, including separate models for butyric acid, propionic acid, acetic acid, and total SCFAs (including butyric acid, propionic acid, acetic acid, isobutyric acid, valeric acid, isovaleric acid, heptanoic acid, and hexanoic acid). We performed a natural log + 1 transformation on butyrate concentrations and propionate at 12 months to normalize the distribution at these timepoints.

After discovering the ASVs significantly associated with timing of complementary foods, we proceeded to calculate Spearman correlations of the relative abundance of these significant ASVs with the major SCFAs (acetic acid, butyric acid, and propionic acid) and total SCFA concentrations in the 3 and 12-month samples.

### Model adjustments and statistical significance thresholds

In addition to timing of complementary feeding (early vs. late), we included breastfeeding (never vs. ever), delivery mode (C-section vs. vaginal delivery), birth weight and gestational age in our multivariable-adjusted models. All multivariable models included birth weight (kg) and gestational age (weeks) as continuous except for the Shannon diversity models, in which we split these covariates at the median to allow model convergence. We adjusted for these potential confounders based on prior literature [13, 31, 70, 71], which indicated these variables were associated with both our exposure and outcome, but were not on the causal pathway. We conducted additional covariate-adjusted analyses to ensure our findings were robust to model adjustment for alternative categorizations of breastfeeding or to model adjustment for infant age at stool sample collection, maternal education, and maternal smoking.

We considered *p* < 0.05 significant for alpha diversity and beta diversity outcome models. To adjust for multiple comparisons in our microbial differential abundance outcomes and SCFA correlations, we defined significance as a two-sided false discovery rate (FDR) adjusted *p* < 0.05.

## Supplementary information

**Additional file 1: Figure S1.** Sequence quality of the forward and reverse 16S rRNA gene reads.

**Additional file 2: Figure S2.** Estimated error rates of the filtered 16S rRNA gene reads.

**Additional file 3: Figure S3.** Summary of sequence counts at each step of the DADA2 processing pipeline.

**Additional file 4: Figure S4.** Weighted UniFrac PCoA plots showing the association of early introduction of complementary food with infant gut microbiota beta diversity at (A) 3 months of age (PERMANOVA beta = 0.02, *p* = 0.26) and (B) 12 months of age (PERMANOVA beta = 0.02, *p* = 0.55). Points are colored by the timing of complementary food introduction and shaped by breastfeeding status (ever vs. never).

**Additional file 5: Table S1.** Differences in the log odds of bacterial ASV relative abundance at 3 months of age accordint to the timing of introduction to complementary foods, after adjustment for delivery mode, breastfeeding, gestational age, and birth weight.

**Additional file 6: Table S2.** Differences in the log odds of bacterial ASV relative abundance at 12 months of age according to the timing of introduction to complementary foods, after adjustment for delivery mode, breastfeeding, gestational age, and birth weight.

**Additional file 7: Table S3.** Unadjusted and multivariable-adjusted linear models examining the association of early introduction of complementary foods with the gut microbiota Shannon diversity, with adjustment for additional covariates.

**Additional file 8: Table S4.** Unadjusted and multivariable-adjusted linear models examining the association of early introduction of complementary foods with the concentration of fecal short-chain fatty acids (in μmol/g) at 3 months of age and 12 months of age, with adjustment for additional covariates.

## Data Availability

The datasets used and/or analyzed during the current study are available from the corresponding author on reasonable request.

## Abbreviations

ASV: Amplicon sequence variant
C-section: Cesarean section
FDR: False discovery rate
GTR: Generalized time-reversible
IVR: Interactive voice response
PERMANOVA: Permutational multivariate analysis of variance
SCFA: Short chain fatty acids
