# Supplementary figures and images for: Timing of complementary feeding is associated with gut microbiota diversity and composition and short chain fatty acid concentrations over the first year of life
Source: BMC Microbiol. 2020 Mar 11;20:56. doi: 10.1186/s12866-020-01723-9 (PMC7065329; doi:10.1186/s12866-020-01723-9)

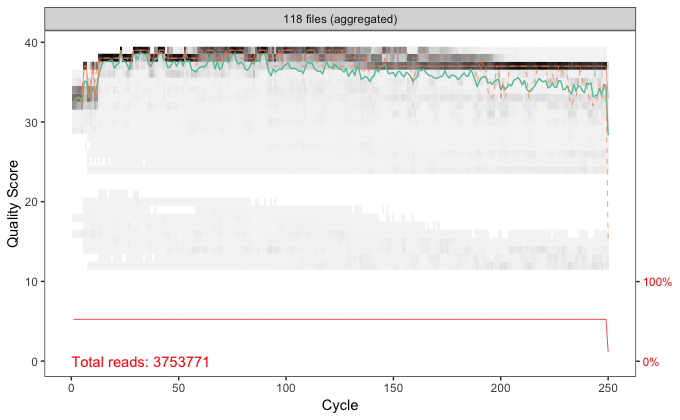


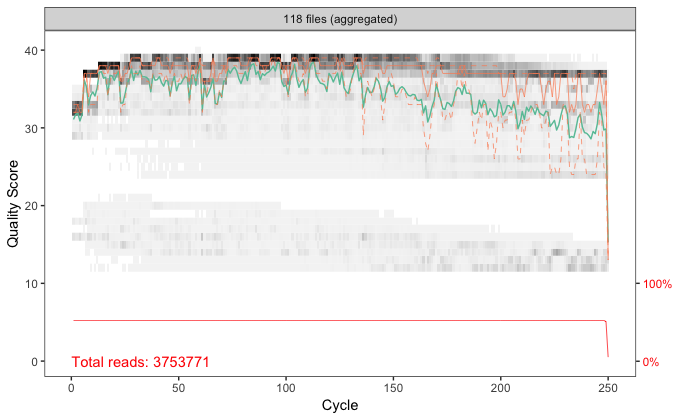

Supplement: Supplementary file 1 — Additional file 1: Figure S1. Sequence quality of the forward and reverse 16S rRNA gene reads. [file 12866_2020_1723_MOESM1_ESM.docx]

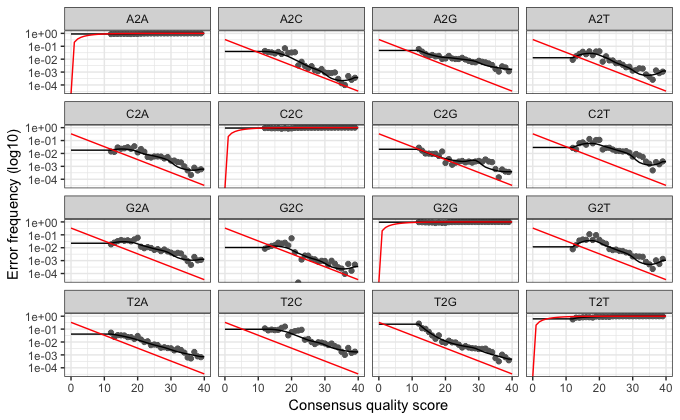


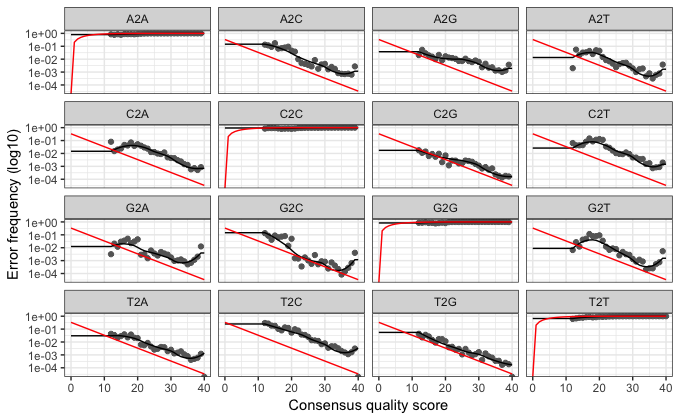

Supplement: Supplementary file 2 — Additional file 2: Figure S2. Estimated error rates of the filtered 16S rRNA gene reads. [file 12866_2020_1723_MOESM2_ESM.docx]

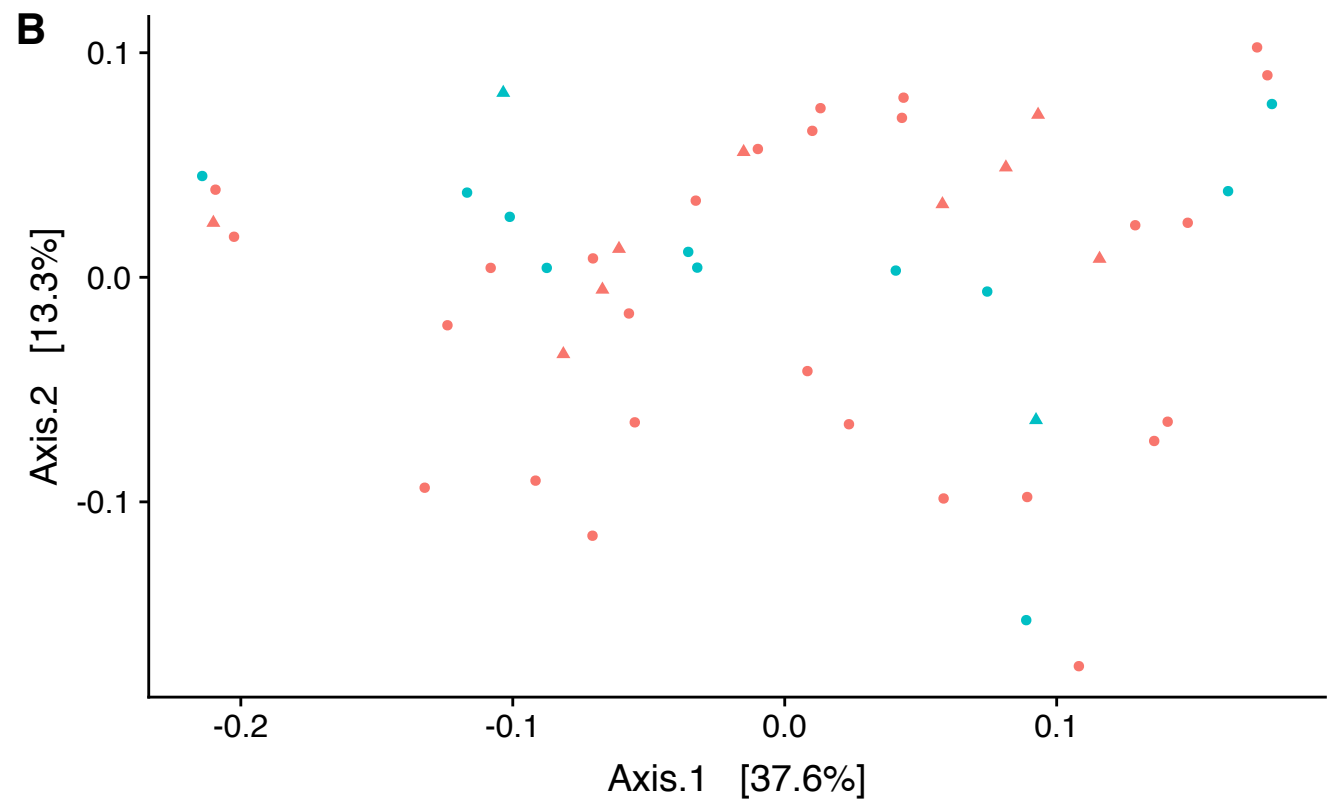

Breastfeeding

- Ever breastfed
- ▲ Never breastfed

Supplement: Supplementary file 4 — Additional file 4: Figure S4. Weighted UniFrac PCoA plots showing the association of early introduction of complementary food with infant gut microbiota beta diversity at (A) 3 months of age (PERMANOVA beta = 0.02, p = 0.26) and (B) 12 months of age (PERMANOVA beta = 0.02, p = 0.55). Points are colored by the timing of complementary food introduction and shaped by breastfeeding status (ever vs. never). [file 12866_2020_1723_MOESM4_ESM.pdf]
