# Supplementary material for: Timing of complementary feeding is associated with gut microbiota diversity and composition and short chain fatty acid concentrations over the first year of life
Source: BMC Microbiol. 2020 Mar 11;20:56. doi: 10.1186/s12866-020-01723-9 (PMC7065329; doi:10.1186/s12866-020-01723-9)
Supplement: Supplementary file 3 — Additional file 3: Figure S3. Summary of sequence counts at each step of the DADA2 processing pipeline. [file 12866_2020_1723_MOESM3_ESM.pdf]

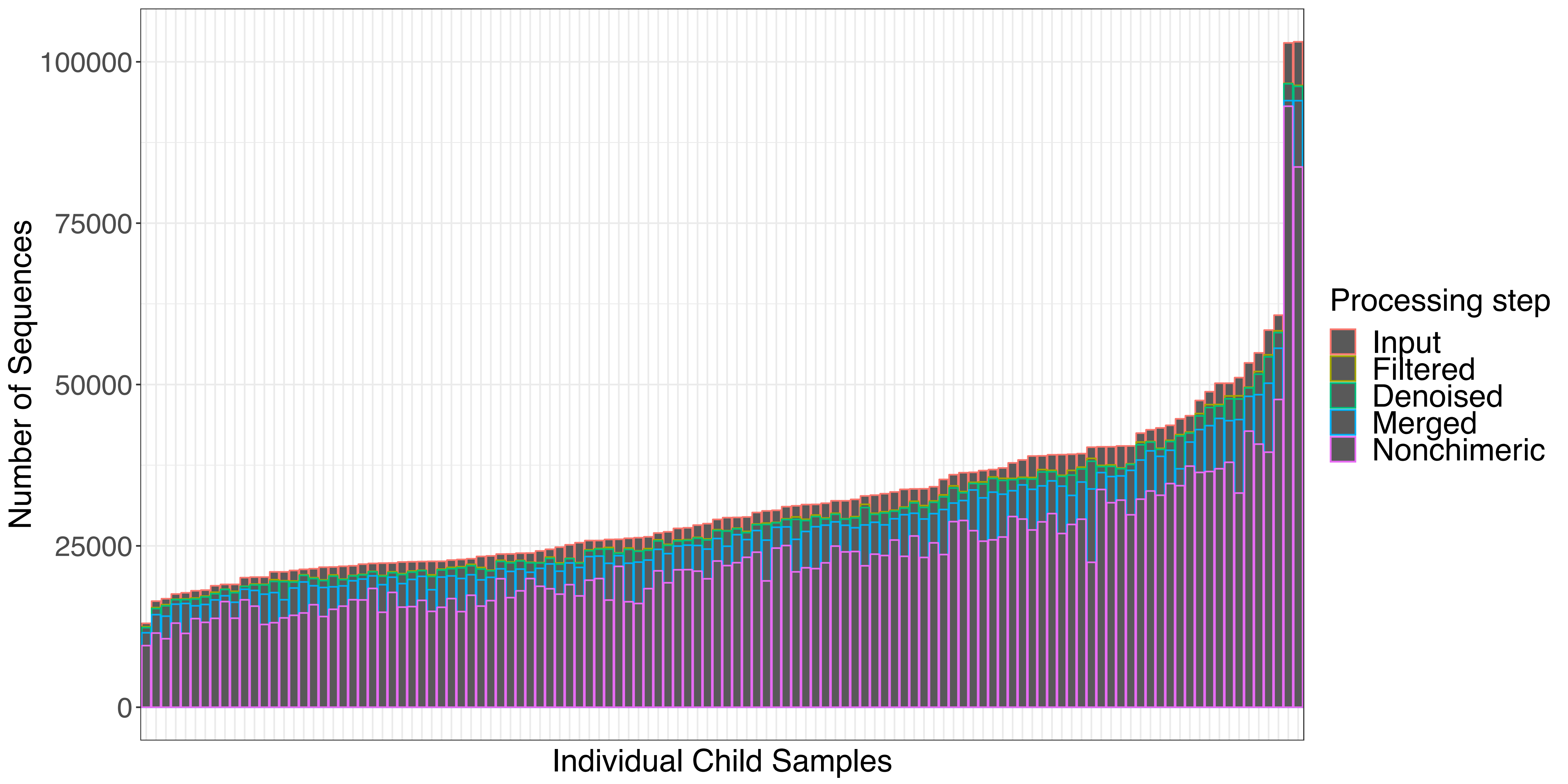
100000
75000
50000
25000
0
Number of Sequences
Individual Child Samples
Processing step
Input
Filtered
Denoised
Merged
Nonchimeric
