# Supplementary material for: Timing of complementary feeding is associated with gut microbiota diversity and composition and short chain fatty acid concentrations over the first year of life
Source: BMC Microbiol. 2020 Mar 11;20:56. doi: 10.1186/s12866-020-01723-9 (PMC7065329; doi:10.1186/s12866-020-01723-9)
Supplement: Supplementary file 5 — Additional file 5: Table S1. Differences in the log odds of bacterial ASV relative abundance at 3 months of age accordint to the timing of introduction to complementary foods, after adjustment for delivery mode, breastfeeding, gestational age, and birth weight. [file 12866_2020_1723_MOESM5_ESM.docx]

Table S1. Differences in the log odds of bacterial ASV relative abundance at 3 months of age according to the timing of introduction to complementary foods, after adjustment for delivery mode, breastfeeding, gestational age, and birth weight.

| Beta | SE | FDR  p-value | Phylum | Class | Order | Family | Genus | Species |
| --- | --- | --- | --- | --- | --- | --- | --- | --- |
| 5.84 | 0.84 | < 0.001 | Verrucomicrobia | Verrucomicrobiae | Verrucomicrobiales | Verrucomicrobiaceae | Akkermansia | muciniphila |
| 3.88 | 0.91 | < 0.001 | Firmicutes | Clostridia | Clostridiales | Lachnospiraceae | Lachnoclostridium | indolis |
| 3.33 | 0.98 | 0.005 | Bacteroidetes | Bacteroidia | Bacteroidales | Bacteroidaceae | Bacteroides | NA |
| 2.52 | 0.9 | 0.024 | Proteobacteria | Gammaproteobacteria | Enterobacteriales | Enterobacteriaceae | Erwinia | NA |
| 1.84 | 0.48 | 0.002 | Firmicutes | Bacilli | Lactobacillales | Streptococcaceae | Streptococcus | NA |
| 1.67 | 0.57 | 0.02 | Firmicutes | Negativicutes | Selenomonadales | Veillonellaceae | Veillonella | NA |
| -1.88 | 0.71 | 0.037 | Firmicutes | Negativicutes | Selenomonadales | Veillonellaceae | Veillonella | NA |
| -2.11 | 0.59 | 0.003 | Proteobacteria | Deltaproteobacteria | Desulfovibrionales | Desulfovibrionaceae | Bilophila | wadsworthia |
| -2.36 | 0.88 | 0.033 | Proteobacteria | Gammaproteobacteria | Enterobacteriales | Enterobacteriaceae | Erwinia | NA |
| -2.4 | 0.84 | 0.022 | Bacteroidetes | Bacteroidia | Bacteroidales | Bacteroidaceae | Bacteroides | NA |
| -2.5 | 0.98 | 0.043 | Actinobacteria | Actinobacteria | Bifidobacteriales | Bifidobacteriaceae | Bifidobacterium | NA |
| -2.6 | 0.83 | 0.011 | Firmicutes | Bacilli | Lactobacillales | Streptococcaceae | Streptococcus | NA |
| -6.1 | 1.73 | 0.004 | Firmicutes | Clostridia | Clostridiales | Veillonellaceae | Dialister | succinicivorans |
