# Supplementary material for: Timing of complementary feeding is associated with gut microbiota diversity and composition and short chain fatty acid concentrations over the first year of life
Source: BMC Microbiol. 2020 Mar 11;20:56. doi: 10.1186/s12866-020-01723-9 (PMC7065329; doi:10.1186/s12866-020-01723-9)
Supplement: Supplementary file 6 — Additional file 6: Table S2. Differences in the log odds of bacterial ASV relative abundance at 12 months of age according to the timing of introduction to complementary foods, after adjustment for delivery mode, breastfeeding, gestational age, and birth weight. [file 12866_2020_1723_MOESM6_ESM.docx]

Table S2. Differences in the log odds of bacterial ASV relative abundance at 12 months of age according to the timing of introduction to complementary foods, after adjustment for delivery mode, breastfeeding, gestational age, and birth weight.

| Beta | SE | FDR  p-value | Phylum | Class | Order | Family | Genus | Species |
| --- | --- | --- | --- | --- | --- | --- | --- | --- |
| 3.62 | 0.39 | < 0.001 | Bacteroidetes | Bacteroidia | Bacteroidales | Porphyromonadaceae | Parabacteroides | NA |
| 2.12 | 0.67 | 0.012 | Firmicutes | Clostridia | Clostridiales | Clostridiaceae | Clostridium | disporicum |
| 1.82 | 0.65 | 0.026 | Firmicutes | Clostridia | Clostridiales | Lachnospiraceae | Roseburia | NA |
| 1.36 | 0.49 | 0.03 | Firmicutes | Negativicutes | Selenomonadales | Veillonellaceae | Veillonella | NA |
| -1.57 | 0.57 | 0.03 | Firmicutes | Clostridia | Clostridiales | Eubacteriaceae | Eubacterium | NA |
| -1.57 | 0.6 | 0.04 | Firmicutes | Clostridia | Clostridiales | Clostridiaceae | Clostridium | paraputrificum |
| -1.71 | 0.41 | 0.001 | Proteobacteria | Gammaproteobacteria | Enterobacteriales | Enterobacteriaceae | NA | NA |
| -2.1 | 0.81 | 0.042 | Firmicutes | Bacilli | Lactobacillales | Enterococcaceae | Enterococcus | NA |
| -2.2 | 0.87 | 0.048 | Firmicutes | Clostridia | Clostridiales | Eubacteriaceae | Eubacterium | hallii |
| -2.6 | 0.69 | 0.003 | Bacteroidetes | Bacteroidia | Bacteroidales | Bacteroidaceae | Bacteroides | uniformis |
| -2.62 | 0.69 | 0.003 | Firmicutes | Clostridia | Clostridiales | Lachnospiraceae | [Ruminococcus] | NA |
| -2.77 | 1.07 | 0.041 | Firmicutes | Negativicutes | Selenomonadales | Veillonellaceae | Veillonella | NA |
| -2.89 | 1.05 | 0.031 | Firmicutes | Clostridia | Clostridiales | Lachnospiraceae | Dorea | formicigenerans |
| -3.64 | 1.01 | 0.004 | Bacteroidetes | Bacteroidia | Bacteroidales | Bacteroidaceae | Bacteroides | NA |
| -3.66 | 1.22 | 0.018 | Bacteroidetes | Bacteroidia | Bacteroidales | Porphyromonadaceae | Parabacteroides | NA |
| -3.85 | 1.43 | 0.035 | Bacteroidetes | Bacteroidia | Bacteroidales | Rikenellaceae | Alistipes | NA |
| -3.96 | 1.53 | 0.042 | Firmicutes | Clostridia | Clostridiales | Lachnospiraceae | Lachnoclostridium | NA |
| -4.02 | 1.34 | 0.018 | Firmicutes | Clostridia | Clostridiales | Lachnospiraceae | Lachnoclostridium | NA |
| -4.12 | 0.98 | 0.001 | Firmicutes | Clostridia | Clostridiales | Eubacteriaceae | Eubacterium | hallii |
| -4.13 | 0.72 | < 0.001 | Firmicutes | Clostridia | Clostridiales | Lachnospiraceae | Lachnoclostridium | indolis |
