# Supplementary material for: Timing of complementary feeding is associated with gut microbiota diversity and composition and short chain fatty acid concentrations over the first year of life
Source: BMC Microbiol. 2020 Mar 11;20:56. doi: 10.1186/s12866-020-01723-9 (PMC7065329; doi:10.1186/s12866-020-01723-9)
Supplement: Supplementary file 7 — Additional file 7: Table S3. Unadjusted and multivariable-adjusted linear models examining the association of early introduction of complementary foods with the gut microbiota Shannon diversity, with adjustment for additional covariates. [file 12866_2020_1723_MOESM7_ESM.docx]

Table S3. Unadjusted and multivariable-adjusted linear models examining the association of early introduction of complementary foods with the gut microbiota Shannon diversity, with adjustment for additional covariates.

| **Supplemental Table 3.** Mean difference (95% CI) for Shannon diversity between infants exposed to complementary foods ≤ 3 months results vs. infants exposed to complementary foods >3 months (reference group), after additional adjustment for reviewer requested covariates. | | |
| --- | --- | --- |
|  | Shannon diversity | |
|  | Crude | Adjusted |
| Model | **Infants at 3 months [Complementary foods ≤ 3 months (n=18)]** | |
| 1 | 0.28*** (0.18, 0.38) | 0.22*** (0.15, 0.30) |
| 2 | - | 0.24** (0.06, 0.42) |
| 3 | - | 0.20** (0.08, 0.32) |
| 4 | - | 0.19* (0.03, 0.34) |
| 5^#^ | - | 0.21* (0.02, 0.39) |
| 6 | - | 0.25** (0.10, 0.41) |
| Model | **Infants at 12 months [Complementary foods ≤ 3 months (n=13)]** | |
| 1 | 0.37** (0.16, 0.58) | 0.31** (0.16, 0.46) |
| 2 | **-** | 0.25** (0.08, 0.43) |
| 3 | **-** | 0.25*  (0.06, 0.44) |
| 4 | **-** | 0.28* (0.06, 0.50) |
| 5^#^ | **-** | 0.52*** (0.32, 0.72) |
| 6 | **-** | 0.36** (0.15, 0.56) |
| * = p < 0.05, ** = p < 0.01, *** = p < 0.001  ^#^7 missing maternal smoking for 3-month microbiome outcomes; 6 missing maternal smoking for 12-month outcomes  All multivariable models adjusted for delivery mode, gestational age, and birth weight.  Model 1 further adjusts for breastfeeding (ever vs. never).  Model 2 adjusts for breastfeeding (duration in weeks, continuous)  Model 3 adjusts for breastfeeding (ever vs. never) in addition to breastfeeding status at the time of first sample collection (still breastfeeding vs. formula only).  Model 4 adjusts for breastfeeding (ever vs. never) as well as age at the time of sampling.  Model 5 adjusts for breastfeeding (ever vs. never) as well low maternal educational achievement (yes vs. no) and current maternal smoking (yes vs. no).  Model 6 adjusts for breastfeeding and antibiotic use (any vs. none) up to sample time point. | | |
