# Supplementary material for: Timing of complementary feeding is associated with gut microbiota diversity and composition and short chain fatty acid concentrations over the first year of life
Source: BMC Microbiol. 2020 Mar 11;20:56. doi: 10.1186/s12866-020-01723-9 (PMC7065329; doi:10.1186/s12866-020-01723-9)
Supplement: Supplementary file 8 — Additional file 8: Table S4. Unadjusted and multivariable-adjusted linear models examining the association of early introduction of complementary foods with the concentration of fecal short-chain fatty acids (in μmol/g) at 3 months of age and 12 months of age, with adjustment for additional covariates. [file 12866_2020_1723_MOESM8_ESM.docx]

Table S4. Unadjusted and multivariable-adjusted linear models examining the association of early introduction of complementary foods with the concentration of fecal short-chain fatty acids (in µmol/g) at 3 months of age and 12 months of age, with adjustment for additional covariates.

| **Supplemental Table 4.** Mean difference (95% CI) for SCFA concentrations (µmol/g) between infants exposed to complementary foods ≤ 3 months results vs. infants exposed to complementary foods >3 months (reference group), after additional adjustment for reviewer requested covariates | | | | | | | | |
| --- | --- | --- | --- | --- | --- | --- | --- | --- |
|  | Butyric Acid^±^ | | Propionic Acid^±^ | | Acetic Acid | | Total SCFAs | |
|  | Crude | Adjusted | Crude | Adjusted | Crude | Adjusted | Crude | Adjusted |
| Model | **Infants at 3 months [Complementary foods ≤ 3 months (n=18)]** | | | | | | | |
| 1 | 0.19  (-0.26, 0.64) | 0.11 (-0.31, 0.53) | 0.01 (-0.39, 0.41) | 0.04 (-0.36, 0.44) | 0.29 (-17.4, 18.0) | -0.36 (-18.6, 17.9) | 3.88 (-20.2, 27.9) | 2.82 (-21.8, 27.4) |
| 2 | - | 0.10 (-0.30, 0.51) | - | 0.04 (-0.36, 0.44) | - | -0.12  (-18.4, 18.2) | - | 2.91  (-21.7, 27.5) |
| 3 | - | 0.07 (-0.34, 0.48) | - | 0.02 (-0.38, 0.42) | - | -0.18 (-18.6, 18.3) | - | 2.09 (-22.8, 26.9) |
| 4 | - | 0.11 (-0.31, 0.54) | - | 0.01 (-0.39, 0.42) | - | -0.19 (-18.8, 18.4) | - | 3.26 (-21.8, 28.3) |
| 5^#^ | - | 0.20 (-0.22, 0.61) | - | 0.04 (-0.43, 0.51) | - | -7.18 (-27.8, 13.4) | - | -1.58 (-29.5, 26.3) |
| 6 | - | 0.11  (-0.31, 0.53) | - | 0.04 (-0.36, 0.45) | - | 0.60 (-17.3, 18.5) | - | 3.83 (-20.6, 28.2) |
| Model | **Infants at 12 months [Complementary foods ≤ 3 months (n=13)]** | | | | | | | |
| 1 | **0.67*** (0.30, 1.04)** | **0.65** (0.27, 1.04)** | **0.35* (0.03, 0.68)** | 0.31 (-0.03, 0.65) | **21.0* (0.65, 41.4)** | 21.3 (-0.42, 43.0) | **40.5** (11.3, 69.8)** | **38.8* (7.83, 69.7)** |
| 2 | **-** | **0.64** (0.25, 1.03)** | **-** | 0.32 (-0.02, 0.65) | **-** | 21.3 (-0.12, 42.7) | **-** | **38.8* (8.23, 69.4)** |
| 3 | **-** | **0.65** (0.26, 1.04)** | **-** | 0.29 (-0.04, 0.63) | **-** | 21.5 (-0.44, 43.5) | **-** | **38.8* (7.40, 70.1)** |
| 4 | **-** | **0.64** (0.25, 1.03)** | **-** | 0.30 (-0.04, 0.64) | **-** | 21.0 (-1.02, 43.0) | **-** | **37.9* (6.70, 69.2)** |
| 5^#^ | **-** | **0.76** (0.30, 1.22)** | **-** | 0.40 (0.01, 0.80) | **-** | **29.2* (2.11, 56.3)** | **-** | **54.8** (16.5, 93.0)** |
| 6 | **-** | **0.66** (0.26, 1.06)** | **-** | 0.34 (-0.01, 0.70) | **-** | 19.01 (-3.69, 41.7) | **-** | **36.8* (4.29, 69.3)** |
| * = p < 0.05, ** = p < 0.01, *** = p < 0.001  ^±^log+1 transformation used for butyrate and propionate at 3 and 12 months.  ^#^7 missing maternal smoking for 3-month microbiome outcomes; 6 missing maternal smoking for 12-month outcomes  All multivariable models adjusted for delivery mode, gestational age, and birth weight.  Model 1 further adjusts for breastfeeding (ever vs. never).  Model 2 adjusts for breastfeeding (duration in weeks, continuous)  Model 3 adjusts for breastfeeding (ever vs. never) in addition to breastfeeding status at the time of first sample collection (still breastfeeding vs. formula only).  Model 4 adjusts for breastfeeding (ever vs. never) as well as age at the time of sampling.  Model 5 adjusts for breastfeeding (ever vs. never) as well low maternal educational achievement (yes vs. no) and current maternal smoking (yes vs. no).  Model 6 adjusts for breastfeeding and antibiotic use (any vs. none) up to sample time point. | | | | | | | | |
